# Influence of Process Parameters on Grain Size and Texture Evolution of Fe-3.2 wt.-% Si Non-Oriented Electrical Steels

**DOI:** 10.3390/ma14226822

**Published:** 2021-11-12

**Authors:** Xuefei Wei, Alexander Krämer, Gerhard Hirt, Anett Stöcker, Rudolf Kawalla, Martin Heller, Sandra Korte-Kerzel, Lucas Böhm, Wolfram Volk, Nora Leuning, Kay Hameyer, Johannes Lohmar

**Affiliations:** 1 Institute of Metal Forming (IBF), RWTH Aachen University, 52072 Aachen, Germany; xuefei.wei@ibf.rwth-aachen.de (X.W.); alexander.kraemer@ibf.rwth-aachen.de (A.K.); johannes.lohmar@ibf.rwth-aachen.de (J.L.); 2 Institute of Metal Forming (IMF), TU Bergakademie Freiberg, 09596 Freiberg, Germany; anett.stoecker@imf.tu-freiberg.de (A.S.); rudolf.kawalla@imf.tu-freiberg.de (R.K.); 3 Institute for Physical Metallurgy and Materials Physics (IMM), RWTH Aachen University, 52074 Aachen, Germany; heller@imm.rwth-aachen.de (M.H.); korte-kerzel@imm.rwth-aachen.de (S.K.-K.); 4 Chair of Metal Forming and Casting (utg), TU München, 85748 Garching, Germany; lucas.boehm@utg.de (L.B.); wolfram.volk@utg.de (W.V.); 5 Institute of Electrical Machines (IEM), RWTH Aachen University, 52062 Aachen, Germany; nora.leuning@iem.rwth-aachen.de (N.L.); kay.hameyer@iem.rwth-aachen.de (K.H.)

**Keywords:** grain size, texture, hot rolling, cold rolling, final annealing, non-oriented electrical steel, process chain, process parameters

## Abstract

The magnetic properties of non-oriented electrical steel, widely used in electric machines, are closely related to the grain size and texture of the material. How to control the evolution of grain size and texture through processing in order to improve the magnetic properties is the research focus of this article. Therefore, the complete process chain of a non-oriented electrical steel with 3.2 wt.-% Si was studied with regard to hot rolling, cold rolling, and final annealing on laboratory scale. Through a comprehensive analysis of the process chain, the influence of important process parameters on the grain size and texture evolution as well as the magnetic properties was determined. It was found that furnace cooling after the last hot rolling pass led to a fully recrystallized grain structure with the favorable ND-rotated-cube component, and a large portion of this component was retained in the thin strip after cold rolling, resulting in a texture with a low γ-fiber and a high ND-cube component after final annealing at moderate to high temperatures. These promising results on a laboratory scale can be regarded as an effective way to control the processing on an industrial scale, to finally tailor the magnetic properties of non-oriented electrical steel according to their final application.

## 1. Introduction

Non-oriented (NO) electrical steel is widely used to make stators and rotors of electric machines like generators and motors. Their overall efficiency and performance depend strongly on the quality of the NO electrical steel. High quality NO electrical steel provides good magnetic properties, especially easy magnetization and low magnetic losses. These magnetic properties are inextricably linked to the grain size and crystallographic texture of the material. Although the magnetic properties of NO electrical steel are much more isotropic compared to oriented electrical steel, the texture is not entirely random, i.e., it is easier to magnetize in some directions. In general, for a body-centered cubic (bcc) iron crystal, <100> is the ideal direction because it is the easiest to magnetize, while <111> is the hardest [1]. For a polycrystal, the orientation distribution, namely the texture, can be described by the orientation distribution function (ODF) f(g), where each crystallographic orientation is represented by Euler angles (φ_1_, Φ, φ_2_). Due to the intrinsic symmetry during rolling and the crystal symmetry of bcc materials, the ODF can be restricted to the subspace 0° < (φ_1_, Φ, φ_2_) < 90°, and the characteristic texture components after rolling and final annealing can be found in the φ_2_ = 45° section. Fibers, i.e., center lines of continuous orientation tubes in Euler space are usually used to describe these characteristic texture components [2]. The typical cold rolling texture of electrical steel consists of two typical orientation fibers, the α-(<110>//RD) and γ-fiber (<111>//ND) [3]. Many current studies are trying to obtain a high fraction of beneficial ND-cube (<100>//ND), also referred to as λ-fiber, as it aids magnetization [4,5]. To quantify the magnetic quality of a texture, Kestens et al. [6] proposed the *A*-parameter, where the angle *A*_θ_(g) is the minimum angle between the magnetization direction $\vec{M}$ and the closest <100> direction of the respective crystal. Thus, θ gives the angle between RD and $\vec{M}$ and since NO electrical steel is polycrystalline, the integral over the whole orientation distribution ($A_{\theta }=\int f\left(g\right)A_{\theta }\left(g\right)dg$) has to be considered for the final A-Parameter. A small A-parameter indicates a high magnetic quality of the texture, because the <100> directions are closely aligned with the magnetic flux. The texture influence on magnetic properties is especially large at high polarizations, where domains are in a rotation stage. Furthermore, the grain size directly affects the magnetic loss. Small grains cause large hysteresis losses, large grains lead to large excess and local eddy current losses, and the relative share of the respective loss components is affected by the sheet thickness and frequency of the magnetic field [7].

It is of great importance to study the texture and grain size development along the entire process chain, because due to a lack of phase transformation (above 2 wt.-% Si, [8]), both are affected by each process step in the production chain. A typical process chain of fully finished electrical steel sheets includes hot rolling, cold rolling, and final annealing. Various studies on the influence of process parameters on grain size and texture, as well as the influence on the final magnetic properties have been carried out already. These will be discussed below in accordance with the processing sequence mentioned above. However, these studies were conducted for a great variety of electrical steels with either different chemical composition or different preceding processing that can additionally influence the results and make them ambiguous. Thus, a comprehensive study for a fixed material composition with identical preceding processing condition was pursued in this paper.

Mager and Wieting [9] concluded that it is possible to create different textures of technical importance in the same material via varying the hot rolling conditions. Consequently, it is important to consider the hot rolling strategy in detail. Hot rolling temperatures, like reheating temperature, finishing temperature [10], and coiling temperature, have an impact on the hot strip grain size and texture as well as their distribution across the strip thickness [11,12,13]. Especially for materials with a phase transformation, like in the mentioned articles, the impact is remarkable. However, for high silicon grades, the austenite to ferrite transformation does not occur. Nevertheless, temperature control is an effective way to adjust the hot strip grain size also for these grades. Usually, a low finishing temperature results in a smaller grain size in comparison to a higher finishing temperature [14,15,16]. A typical hot strip structure of ferritic electrical steel produced by continuous rolling has small equiaxed grains at the subsurface layer and elongated grains in the mid layer [13,16]. Additionally, a texture gradient between surface and center of the hot strip is present [17]. In most studies, commercially produced hot strip with thicknesses from 2 to 3 mm is used [10,18], whereby no detailed information on the hot rolling parameters is provided. Notwithstanding, specially designed microstructures were achieved by additional hot strip annealing (average grain size of up to 500 µm) [3,19] or unconventional laboratory hot strip processing, such as hot rolling of samples with fully columnar grains [4] or using the hot strip in transverse direction for cold rolling [5]. According to Inagaki [20], cube fiber texture components are stable during cold rolling of iron. As cube fiber texture components improve magnetic properties, it seems to be advantageous to have this texture already in the hot strip of NO electrical steel and find ways to preserve it during final annealing. Another aspect is the impact of the hot rolling conditions on the softening processes during final annealing (recovery and recrystallization) [21]. Moreover, the hot strip thickness defines the total deformation energy required during cold rolling and therefore indirectly the driving force for recrystallization during final annealing, which in turn influences the final grain size, texture, and overall magnetic properties. For example, Paolinelli et al. [15] found the lowest magnetic losses at a cold rolling reduction of 0.64 for a Fe-3 wt.-% Si NO electrical steel by varying the initial hot strip thickness. In summary, the literature suggests that a hot strip with a low thickness of ~1 mm (facilitates cold rolling process) and a pronounced banded structure (high driving force for recrystallization) is beneficial as low cold rolling degrees are required and thus high amounts of cube fiber texture (easy magnetizability) can be preserved. This is hard to achieve for a thicker hot strip with a rather homogeneous grain size distribution and random texture.

Cold rolling determines the final thickness, whilst also changing texture and grain shape to a certain degree. As already mentioned, the stored energy induced by cold rolling affects the texture and grain size evolution during recrystallization and grain growth in the course of final annealing. Therefore, cold rolling parameters, such as reduction degree and number of rolling passes, have a significant influence on the final texture and grain size. As conventional cold rolling typically conserves and even enhances the α- and γ-fiber [2], the potential to control texture via a different stress distribution or temperature range in non-conventional rolling strategies like cross rolling [22,23], reverse rolling [24,25], warm rolling [19,26], and asymmetric rolling [18,27] has been studied. For example, Zhang et al. [26] investigated warm rolling (initial temperature of 300 °C) for a Fe-2.5 wt.-% Si-0.52 wt.-% Al NO electrical steel and concluded that warm rolling increases the nucleation of favorable η-fiber (<001>//RD) nuclei and results in an overall stronger η-fiber and weaker γ-fiber at moderate strain. However, after studying a Fe-3.0 wt.-% Si NO electrical steel, Lee et al. [19] found that a larger volume fraction of ND-cube was generated after warm rolling at 300 °C and final annealing, in addition to some GOSS texture. In conclusion, beneficial texture components can be evoked by non-conventional rolling strategies paired with proper process parameters. However, as these studies used different materials, a general statement concerning the effectiveness of each strategy compared to other strategies cannot be derived. In order to achieve such a comparison, a comprehensive study was conducted here using the same material for all investigations but only considering conventional cold rolling to keep the number of variables as small as possible.

During final annealing of cold rolled thin sheet, the microstructure is reconstructed through recovery and recrystallization. Subsequently, grain growth further shapes the microstructure and final texture. In the course of recrystallization, the microstructure is fully reconstructed through the generation and movement of high angle grain boundaries, which is also related to as nucleation and growth. The main driving force for recrystallization is the difference in stored energy, represented by the dislocation density, between nuclei and the deformed microstructure [28,29]. This initial density varies depending on the rolling conditions (temperature, rolling degree, etc.), as well as the grain orientation relative to the imposed force [30]. Moreover, shear/deformation/transition bands are thought to have the highest dislocation density [31]. As a driving force, the dislocation density determines the recrystallization kinetics and indirectly also the final grain size. Two other important factors are the annealing temperature and time. The higher the temperature (and dislocation density), the higher the nucleation and growth rate, whereby in most cases, the nucleation rate increases faster than the growth rate with increasing temperature [32]. However, if the nucleation rate is too high, the nuclei touch each other earlier, which can obstruct further fast growth because the change from the recrystallization regime to the grain growth regime takes place earlier [31]. During grain growth, the total energy is minimized through the reduction in grain boundary surface area, which is a much smaller driving force than that of recrystallization. The kinetics mainly depend on individual grain boundary and triple junction mobilities [33]. Higher temperatures heavily increase the grain boundary motilities and thus the recrystallization and grain growth kinetics. Moreover, the maximum possible grain size is limited by the annealing temperature, which in turn can be attributed to the thermal activation of grain boundary movements, whereby solute drag and grain-boundary roughening play an important role [34]. Again, the higher the temperature, the higher the possible grain size. For example, Mehdi et al. [35] found a grain size of ~82 µm after annealing a Fe-3.2 wt.-% Si-0.58 wt.-% Al-0.4 wt.-% Mn steel at 750 °C for 180 min and ~187 µm after annealing at 1150 °C for 2 min; Fang et al. [36] found a grain size of ~100 µm after annealing a Fe-1.5 wt.-% Si steel at 950 °C for 10 min; and Pedrosa et al. [37] found a grain size of ~50 µm after annealing a Fe-3.4 wt.-% Si-0.59 wt.-% Mn steel at 850 °C for 30 s and 212 µm after annealing at 1050 °C for 30 s. From these data, it can again be seen that more parameters such as the chemical composition and prior processing have an influence on the final grain size. Additionally, there seems to be temperatures for specific alloys where the grain boundary mobility/energy distribution and segregation behavior (slowing down certain grain boundaries) is so diverse that abnormal grain growth occurs [34]. Usually, the texture of standard NO electrical steel is very similar after final annealing and its evolution is closely linked to the rolling texture. Starting with a relatively strong α- and γ-fiber (mainly near the surface because of shear forces) after rolling, the α-fiber dissolves completely, as well as most of the γ-fiber, and the ND-rotated cube component becomes weaker and often moves towards the cube component. Instead, a high intensity around the Euler angles φ_1_ = 18°, ϕ = 39°, φ_2_ = 45° and φ_1_ = 90°, ϕ = 63°, φ_2_ = 45° as well as a weak component around ND-rotated cube to cube is formed [17].

While a general understanding of process parameters influencing the microstructure and texture evolution in NO electrical steels can be derived from current literature, a comprehensive study that focuses on industrially relevant processing parameters applied throughout the complete processing chain while keeping the chemical composition fixed is lacking. Such a study will be presented in the present paper, which is the result of a collaboration within the research group FOR1897 “Low-Loss Electrical Steel Sheet for Energy-Efficient Electrical Drives”. While the research group also considered a NO electrical steel with a silicon content of 2.4 wt.-%, in this study, a 3.2 wt.-% Si NO electrical steel often used commercially was considered. This steel was chosen as higher silicon contents benefit the magnetic properties while a silicon content above 3.2 wt.-% can cause brittleness during cold rolling [38].

In the present study, two hot strips with identical chemical composition but different microstructures (grain size and structure) and textures were produced by altering selected hot rolling parameters. Furthermore, the influence of the resulting two hot strip states on cold rolling and final annealing is discussed. Additionally, the strips were cold rolled to two different final thicknesses in order to also study the influence of cold rolling degree on final grain size and texture. This aimed to trial a general rule applied in industry, which implies that thin strip generally results in better magnetic properties. Furthermore, annealing was conducted at various temperatures in order to find guidelines for controlling the grain size and optimizing the texture. Such a detailed analysis of all process parameters as well as microstructures and textures along the process chain has not been conducted for Fe-3.2 wt.-% Si NO electrical steel, yet. Still such study is important since on the one hand, every process step has an influence on the final properties and on the other hand, processing is sensitive to the material considered. Thus, a comprehensive analysis is only possible by considering the same material throughout. At the same time, the applicability of the obtained knowledge was tested and if suitable, the results were compared to previous studies on Fe-2.4 wt.-% Si NO electrical steel to assess if the process parameter influence is also dependent on the silicon content. Through a comprehensive study of the influence of different process parameters along the process chain on the evolution of texture and grain size, the targeted adjustment of magnetic properties becomes feasible.

## 2. Materials and Methods

As mentioned above the material investigated in this study was a common NO electrical steel with a silicon content of 3.2 wt.-%. The chemical composition was determined via optical emission spectroscopy and is given in Table 1.

**Figure 1:**
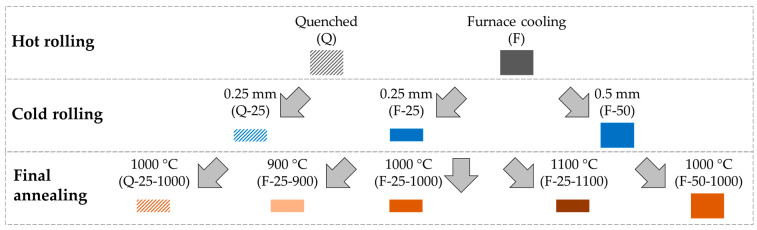
Experimental matrix considering relevant parameters along the process chain.

In this study, the whole processing chain was considered and as different states concerning relevant parameters were investigated, a tree-like structure branching at all process steps as shown in Figure 1 arose. Two kinds of hot strip states were investigated, one obtained by water quenching and the other by furnace cooling after rolling. Both cooling methods are often used in industry. However, the hot strip thickness of 1 mm was thinner compared to conventional production to reduce the total reduction required during cold rolling. Two final thicknesses were considered in cold rolling, 0.25 mm and 0.50 mm, which are common thicknesses for commercial NO electrical steel. Finally, three temperatures were used in the final annealing process, namely 900 °C, 1000 °C, and 1100 °C. At all these temperatures, the sheet can recrystallize completely, eliminating the negative effect of dislocations on the magnetic properties, which were initially introduced during rolling. The number of investigations was kept as small as possible while still enabling the assessment of all parameters that are particularly important to the respective process steps. Consequently, not every possible parameter combination was investigated. Next, each process step along the process chain is introduced in detail.

**Hot rolling** was conducted on a laboratory four-stand semi-continuous hot rolling mill whereby the first stand was used as a reversing breakdown stand while only the second to fourth stand acted as a continuous rolling line. It is also possible to combine the rolling stands to achieve four continuous rolling passes, as was done in this study. Table 2 represents the pass schedule for the hot rolling trials. The 3.2 wt.-% Si feedstock of a 34 mm industrially produced transfer bar was annealed in an argon atmosphere for 30 min at 1300 °C to achieve a homogeneous grain size. Before hot rolling, the material was reheated to 1050 °C. After a soaking time of 20 min, the hot rolling starting temperature was 1030 °C. For the hot strip with a deformed microstructure (Q), the final rolling temperature was 850 °C followed by water quenching. In contrast, for the hot strip with a fully recrystallized microstructure (F), the final rolling temperature was 880 °C followed by slow furnace cooling with 50 K/h to room temperature.

**Cold rolling** was conducted on a four-high rolling mill. The hot strip with a thickness of 1.0 mm was rolled at room temperature to 0.50 mm in three passes and to 0.25 mm in six passes, using the pass schedule shown in Table 3. The actual thickness was within 5% of the target thickness. The diameter of the work rolls was 150 mm and a relatively low rolling speed of 0.05 m/s was used to prevent thermal stresses caused by deformation heat that cannot be dissipated fast enough. The two thicknesses 0.50 mm and 0.25 mm served to investigate the influence of the cold rolling degree on texture and grain size evolution.

**Final annealing** was conducted in a furnace in which strip tension could be applied to the samples. This furnace was specially designed for annealing electrical steel based on preliminary research. Typically, on the laboratory scale, if a short (~500 mm) cold rolled electrical steel sample is heated in a conventional furnace (no strip tension), the sample becomes uneven, probably due to residual and thermal stresses. This is especially true for high temperatures (≥1000 °C) and thin strips (≤0.25 mm). The furnace with strip tension is similar to a continuous heat treatment furnace on the industrial scale where coiling creates the strip tension. By applying a small amount of tension during the annealing process, the sample is prevented from buckling. The required tension varies with annealing temperature and strip thickness. In our experiments, around 80% of the yield stress at the respective annealing temperatures gave the best results. The applied tension should be below the yield stress to prevent plastic deformation. Additionally, the cold rolled strips were wrapped in metal foil to protect them from surface oxidation. Three different annealing strategies were applied: 900 °C/120 s, 1000 °C/60 s, and 1100 °C/60 s. To ensure full recrystallization, the annealing time at 900 °C was 60 s longer than that at 1000 °C and 1100 °C. Finally, the samples were cooled to room temperature in air under tension. Table 4 summarizes all sample states after final annealing, corresponding to the bottom row of the tree structure shown in Figure 1.

**Grain size** was measured using the mean linear intercept method. The micrographs were taken from two planes: RD-ND (Rolling Direction–Normal Direction) and RD-TD (Rolling Direction–Transverse Direction). All measurements in the RD-TD plane were taken in two layers: near the surface and in the mid layer. For the RD-TD micrographs near the surface, the samples were just slightly ground; for the RD-TD mid layer micrographs, the samples were ground to 50% of their total thickness. The final surface finish before etching with 5% Nital was achieved with a 1 µm alcohol-based diamond suspension. In both cases, more than 300 grains were measured to ensure reliable grain size distribution and mean grain size values when using the mean linear intercept method.

**Texture** was measured by XRD (X-ray diffraction) using a Bruker D8 diffractometer equipped with a high resolution area detector. Similar to the grain size, the texture was measured in two layers, near the surface and in the mid layer. The data of three incomplete pole figures: {011}, {002}, and {112} were collected in an area of 8 mm * 8 mm (sample dimensions RD * TD: 12 mm * 10 mm) using filtered Fe-K_α_ radiation at 30 kV and 25 mA. Finally, the orientation distribution function (ODF) was calculated from the incomplete pole figures using the MATLAB toolbox “MTEX” [39].

The **A-Parameter** was calculated based on the XRD texture results using the methodology put forward by Kestens et al. [6] and already introduced above (Section 1). In this context, a MATLAB script utilizing “MTEX” was written, which formed the integral over all texture components and the respective minimum deviation angle of an “easy” <100> direction to a theoretical magnetization vector. Therefore, for θ = 0° (x-axis), this magnetization vector overlapped with RD and for θ = 90° with TD. Hence, starting from a mean value (30°), a strong cube texture reduced the A-parameter and α- as well as γ-components increasing its value.

## 3. Results

As already shown in Figure 1, the process chain for electrical steel consists of three main steps, namely hot rolling, cold rolling, and final annealing. For each of these three steps, experimental investigations were carried out to identify the influence of the process parameters introduced in detail above on both the microstructure in terms of grain size distribution and the texture in terms of ODFs. In addition, for the final annealed state, the texture influence on the magnetic properties was estimated in terms of the aforementioned A-Parameter. A detailed comparison of the A-parameter estimate to measured magnetic properties is not part of this study but is instead given in a recent publication by some of the present authors [40].

### 3.1. Hot Rolling

**Figure 2:**
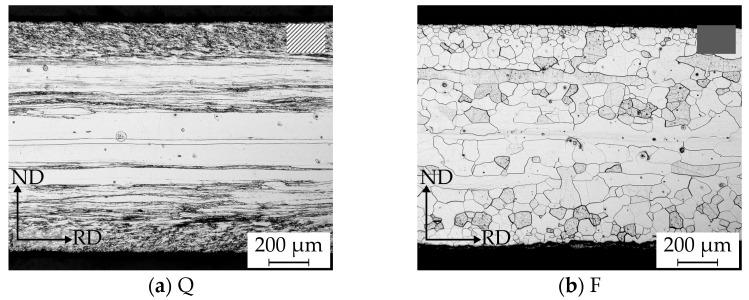
RD-ND micrographs of two hot strip states resulting from different cooling strategies: (**a**) hot strip Q after water quenching; (**b**) hot strip F after furnace cooling.

As Figure 2 shows, the finishing temperature as well as the cooling conditions after the last hot rolling pass affected the hot strip microstructure significantly. A low finishing temperature in combination with water quenching after hot rolling (hot strip Q, subfigure a) led to a deformed state with small grains (mean lineal intercept length of 6.52 ± 4.87 µm) near the surface and a banded structure with flat elongated grains in the mid layer, whereas a furnace cooling after hot rolling (hot strip F, subfigure b) resulted in a fully recrystallized, more homogeneous microstructure across the strip thickness with a mean grain size of 46.85 ± 29.25 µm near the surface and 100.85 ± 49.95 µm in the mid layer.

**Figure 3:**
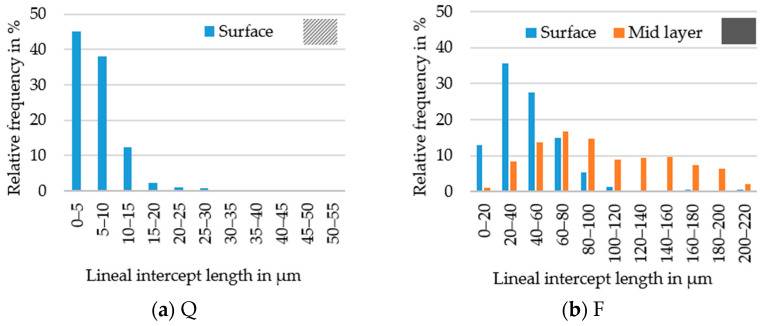
Grain size histograms in the mid and surface layer: (**a**) for hot strip Q where grain size measurements in the mid layer were impossible, (**b**) for hot strip F.

Aside from the micrographs of the hot strips, Figure 3 shows the grain size distribution. For hot strip Q in Figure 3a, successful grain size measurements were only possible in the area near the surface, resulting in a narrow grain size distribution with few grains larger than 30 µm. The grain size distribution of the recrystallized microstructure of hot strip F is shown in Figure 3b. Near the surface as well as in the mid layer, a unimodal positively skewed grain size distribution exists. The grains in the mid layer were about twice the size of the grains near the surface, also showing single grains larger than 200 µm. However, individual grains in the mid layer still showed a banded structure.

**Figure 4:**
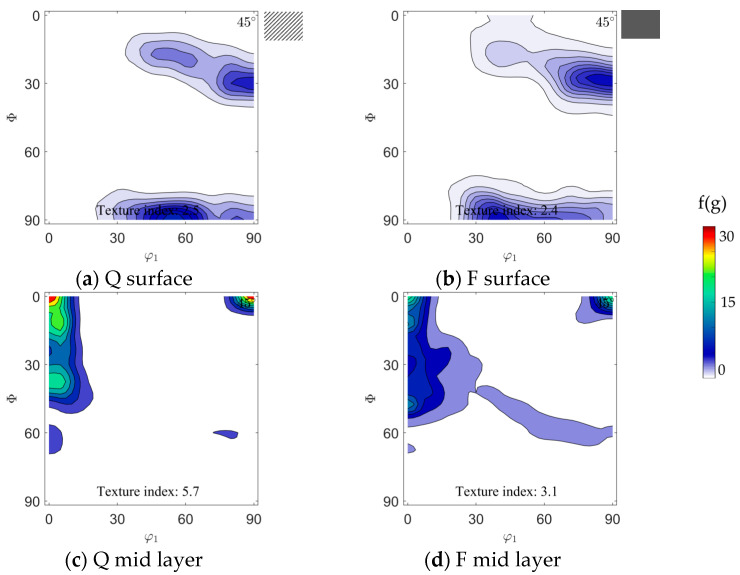
Textures near the surface and in the mid layer of hot strip Q and F: Water-quenched hot strip Q (**a**) near the surface and (**c**) in the mid layer; furnace-cooled hot strip F (**b**) near the surface and (**d**) in the mid layer. Textures are visualized for the ODF φ_2_ = 45° section.

In agreement with the grain size gradient, a strong texture gradient between surface and mid layer could also be found in the hot strips, see Figure 4. Therefore, the main texture components were similar for the two hot strips, yet strip F showed a weaker texture than strip Q. At areas near the surface, pronounced orientations between φ_1_ = 55°, ϕ = 90°, φ_2_ = 45° and Goss φ_1_ = 90°, ϕ = 90°, φ_2_ = 45° as well as φ_1_ = 90°, ϕ = 27°, φ_2_ = 45° and φ_1_ = 90°, ϕ = 35°, φ_2_ = 45° were present. In the mid layer, generally, a strong α-fiber was observed with a maximum at ND-rotated-cube, associated with the banded structure. For hot strip F, additionally, a weak intensity along the γ-fiber was present while hot strip Q showed a higher intensity along the α-fiber with a peak at φ_1_ = 0°, ϕ = 40°, φ_2_ = 45°.

### 3.2. Cold Rolling

**Figure 5:**
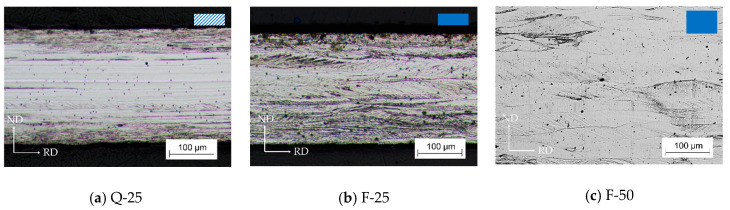
Micrographs of the three cold rolled strips resulting from two hot strip states and two cold rolling pass schedules: (**a**) 0.25 mm cold strip Q-25 after 6 cold rolling passes of hot strip Q; (**b**) 0.25 mm cold strip F-25 after 6 cold rolling passes of hot strip F; (**c**) 0.50 mm cold strip F-50 after 3 cold rolling passes of hot strip F.

Cold rolling defines the final thickness and increases the driving force for recrystallization during final annealing. This driving force is directly coupled to deformation and the stored energy. Because of this, apart from the hot strip state (Q or F), the cold rolling reduction also influences the grain size and texture evolution. Thus, these two parameters were studied for three different cold rolled strips as shown in Figure 1. The micrographs of these three investigated cold strips are shown in Figure 5. Since the grains were largely elongated and the grain boundaries were hard to differentiate in most cases, precise grain size measurements after cold rolling were impossible and instead a qualitative description of the grain size evolution during cold rolling is given in this section.

#### 3.2.1. Influence of Hot Strip Microstructure

In Section 3.1, two hot strips with different cooling strategies were introduced that showed different microstructure and texture. This section shows how these two different hot strip states affected the microstructure and texture after cold rolling. To eliminate the influence of other factors, such as final thickness and cold rolling schedule, these two hot strips Q and F were cold rolled to a final thickness of 0.25 mm according to an identical pass schedule (see Table 3). The final thickness of 0.25 mm was chosen because there is a general trend towards thinner strips in industry because thinner strips generally possess lower eddy current loss.

Figure 5a,b show the cold strips Q-25 based on the water quenched hot strip and F-25 based on the furnace cooled hot strip. After cold rolling, elongated grains and a banded structure were present in both cases. For Q-25, the dominant band in the mid layer of the hot strip was preserved during cold rolling. As can be seen in the micrograph in Figure 5a, only horizontal grain boundaries between different bands were visible by optical light microscopy. Related investigations by EBSD measurements [21] showed the absence of grain boundaries in such banded structures and therefore these areas are assumed to possess a low stored energy. In contrast, after cold rolling of hot strip F, the initial grains were still visible but strongly elongated with deformation bands in most grains, Figure 5b.

**Figure 6:**
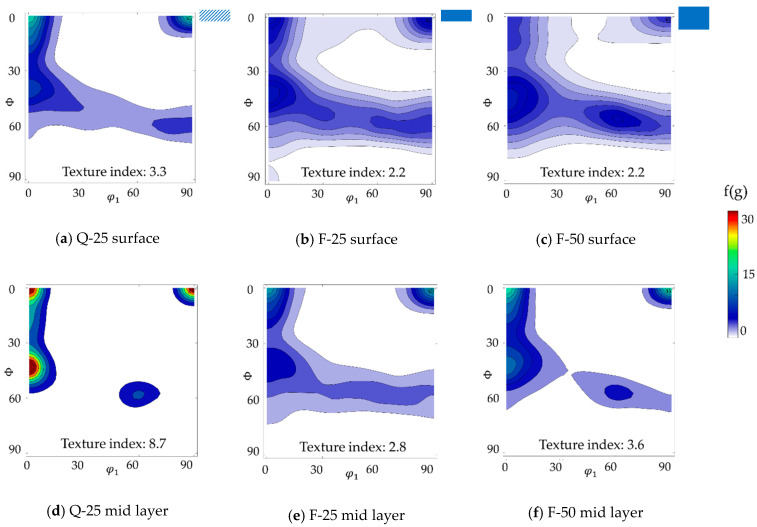
Texture near the surface and in the mid layer of cold strips Q-25, F-25, and F-50: (**a**) Q-25 near the surface; (**b**) F-25 near the surface; (**c**) F-50 near the surface; (**d**) Q-25 in the mid layer; (**e**) F-25 in the mid layer; (**f**) F-50 in the mid layer. Textures are displayed using the ODF φ_2_ = 45° section.

Concerning the texture, the intensity of the α- and γ-fiber increased during cold rolling as expected. Depending on the hot strip textures, the cold rolling textures in Figure 6a,b,d,e reveal different characteristics. For Q-25, a dominant rotated ND-cube texture as well as a peak at φ_1_ = 0°, ϕ = 40°, φ_2_ = 45° could be found. The components became even more dominant in the mid layer after cold rolling (Figure 6d) and were also stronger in the surface layer as compared to F-25 (Figure 6a,b).

#### 3.2.2. Influence of Thickness Reduction

As mentioned in the beginning of this section, the final thickness is determined by cold rolling. Different thickness reductions can affect the evolution of grain size and texture during cold rolling, even for similar hot strips. In this section, the influence of the cold rolling reduction and thus the final thickness on the cold rolled grain structure and texture is presented. The hot strip F mentioned in Section 3.1 is considered here as it had moderate ND-rotated-cube texture component in the mid layer without a peak close to the γ-fiber (φ_1_ = 0°, ϕ = 40°, φ_2_ = 45°), thus providing a promising state for texture optimization. Furthermore, the grain size distribution of hot strip F was more homogeneous and free of bands (see Figure 2) when compared to hot strip Q. The 1 mm strip was cold rolled to 0.25 mm and 0.50 mm based on the pass schedule in Table 3. The micrographs of the 0.25 mm cold strip F-25 and the 0.50 mm cold strip F-50 are shown in Figure 5b,c respectively. Since the hot strip F had a fully recrystallized microstructure, as shown in Figure 2b, vertical grain boundaries could still be seen after cold rolling. In the thinner strip, the grains were more elongated in the rolling direction and more shear/deformation bands occurred.

Figure 6b,c,e,f show the textures near the surface and in the mid layer of the two cold strips F-25 and F-50. The textures after cold rolling were very similar in both cases. Near the surface, the orientations between φ_1_ = 55°, ϕ = 90°, φ_2_ = 45° and Goss φ_1_ = 90°, ϕ = 90°, φ_2_ = 45° as well as φ_1_ = 90°, ϕ = 27°, φ_2_ = 45° and φ_1_ = 90°, ϕ = 35°, φ_2_ = 45° were transformed to a typical rolling texture with α- and γ-fiber, whereas the applied cold rolling degrees had no obvious influence. In the mid layer, a larger portion of ND-rotated-cube was preserved after cold rolling.

### 3.3. Final Annealing

**Figure 7:**
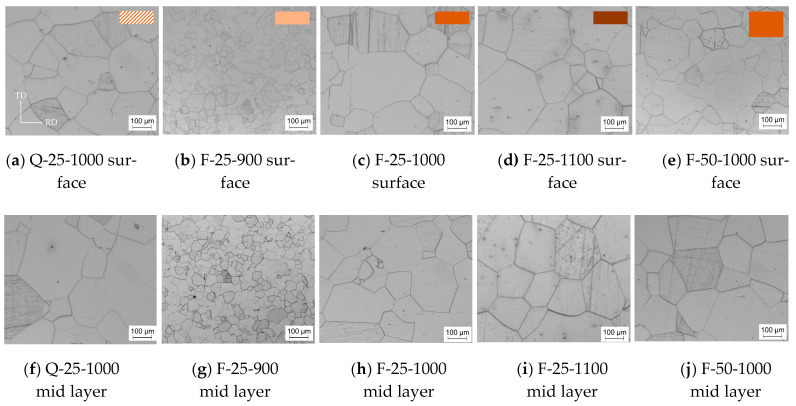
Micrographs near the surface (**a**–**e**) and in the mid layer (**f**–**j**) for annealed sheets: (**a**,**f**) Q-25-1000, (**b**,**g**) F-25-900, (**c**,**h**) F-25-1000, (**d**,**i**) F-25-1100, (**e**,**j**) F-50-1000. All micrographs were taken in the RD-TD plane.

The annealing process determines both the final grain size and texture via recovery, recrystallization, and grain growth. Out of all annealing parameters, the temperature has the strongest influence on the grain size and texture development. This relationship was investigated in detail by heat treating F-25 at three different temperatures: 900 °C for 120 s, 1000 °C for 60 s, and 1100 °C for 60 s (Figure 7b–d,g–i). F-25 was chosen over F-50 and Q-25 because of its texture with a moderate ND-rotated-cube component but without a distinct peak near the γ-fiber and additionally its homogeneous microstructure. Furthermore, the material conditions set in the previous processing steps (Section 3.1 and Section 3.2) can also affect the final grain size and texture. To investigate this in some depth, Figure 7 shows exemplary micrographs of all final annealed sheets (F-25, F-50, and Q-25) corresponding to the last row of Figure 1.

In Section 3.2.1, two cold strips with different final thicknesses were introduced. The degree of deformation during cold rolling can affect the grain size and texture evolution during final annealing. Therefore, two cold rolled strips F-25 and F-50 were annealed at 1000 °C for 1 min. By comparing F-25-1000 and F-50-1000 in Figure 7c,e,h,j, the influence of different cold rolling degrees can be analyzed.

In Section 3.2.2, two cold rolled strips obtained from differently cooled hot strips were introduced. The goal was next to investigate whether this had an effect on grain size and texture evolution during final annealing. By comparing Q-25-1000 and F-25-1000 in Figure 7a,c,f,h, the influence of cold rolled, differently cooled hot strips on the microstructure and texture development during final annealing could be analyzed. Both cold strips (Q-25 and F-25) were annealed at 1000 °C for 1 min.

In the following sections, the influence of temperature, cold rolling degree, and hot band cooling strategy on final grain size, texture, and A-parameter is presented. The A-parameter was considered for annealed sheets only, as it enables an assessment of the final texture in view of the expected magnetic properties [6,41].

#### 3.3.1. Influence on Grain Size

**Figure 8:**
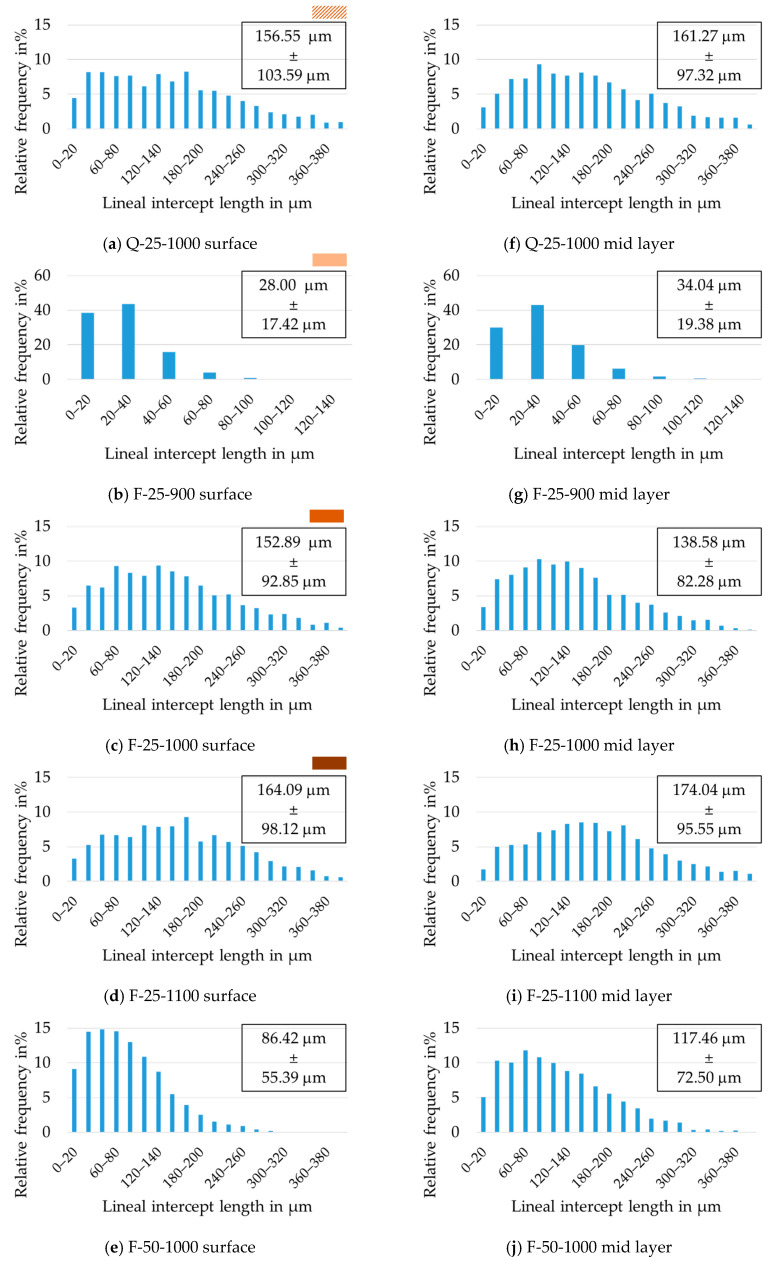
Grain size histograms, averages, and standard deviations near the surface (**a**–**e**) and in the mid layer (**f**–**j**) for annealed sheets: (**a**,**f**) Q-25-1000, (**b**,**g**) F-25-900, (**c**,**h**) F-25-1000, (**d**,**i**) F-25-1100, (**e**,**j**) F-50-1000. All measurements were done according to the line intercept method.

The grain size distribution of all annealed sheets is summarized in Figure 8, while the mean grain size is additionally given in Table 5 for easier comparison. For all sheets the results are given in the mid layer and near the surface.

The influence of the annealing temperature on the grain size distribution is shown in Figure 8b–d,g–i. The average grain size increased with increasing temperature. A temperature of 900 °C resulted in the smallest grain size of 28 µm measured in the surface layer of the annealed sheet (Figure 8b) and 1100 °C in the highest of 174 µm (Figure 8i) measured near the surface. Generally, a trend towards larger grain sizes for the respective mid layers could be seen across all heat treatments, whereby Figure 8h was an outlier. However, this outlier was well within the standard deviation. The standard deviation was always about 60% of the average grain size value, even when the annealing temperature increased, therefore the grain size distribution got broader. All distributions were asymmetric and extended towards larger grain sizes, resulting in typical log-normal distributions. The maximum possible average grain size seemed to be limited for the F-25 sample, because annealing at 1100 °C (F-25-1100) did not show higher averages than annealing at 1000 °C (F-25-1000); however, a higher fraction of very large grains (>350 µm) was present in the former. The maximum grain size can possibly be correlated to the small sheet thickness and free surface effects [32] as well as a metastable thermodynamic equilibrium for the given temperatures. Beyond that, annealing at 1000 °C as well as 1100 °C seemed to yield bimodal distributions with a small second peak developing at very high grain sizes (300–375 µm), which is an indication of abnormal grain growth.

The influence of different cold rolling degrees on grain size after final annealing at 1000 °C is shown in Figure 8c,e,h,j. In all cases, the grain size distribution was very wide. This indicates that the grains passed the recrystallization regime and already experienced some degree of grain growth. Nucleation and growth are affected by microstructure and driving force; therefore, grains of different size appeared for F-25-1000 and F-50-1000. The average grain size of the thinner sheet was larger than that of the thicker sheet, irrespective of through thickness position. This phenomenon is counterintuitive. In general, larger deformation produces more nuclei and a higher dislocation density. The latter is equivalent to a higher stored energy and thus a higher driving force. In particular, more nuclei should lead to a smaller grain size because the nuclei touch each other earlier and further growth is obstructed. In this study, at an annealing temperature of 1000 °C, the thinner sheet possessed a larger grain size. This might be due to a higher driving force in the thinner sheet, greatly accelerating recrystallization and allowing for additional grain growth, resulting in a larger grain size than in the thicker sheet. This is supported by several extremely large grains (above 300 µm) occurring in Figure 8c,h. For the 0.5 mm sheet, the grain size near the surface was smaller than that in the mid layer. The reason could be the additional shear stresses near the surface during cold rolling, which are more conducive to nucleation. However, this phenomenon was not observed in the 0.25 mm sheet. The reason might be that the stress near the surface and in the mid layer were more evenly distributed after cold rolling due to the very thin thickness or the difference equalizes with increasing grain size.

The influence of cold rolled, differently cooled hot strips on grain size after annealing is shown in Figure 8a,c,f,h. During annealing, these two strips behaved differently as the stored energy for recrystallization and the nucleation sites in their respective microstructures were distributed differently across the sheet thickness. Comparing the grain size after annealing for the two initial hot strips Q and F at 1000 °C shows that the grain size of Q-25-1000 with the banded hot strip structure was slightly larger. The grain size distributions were similar. Overall, material Q showed a trend towards a multimodal distribution with the larger variation resulting from selective grain growth.

#### 3.3.2. Influence on Texture

**Figure 9:**
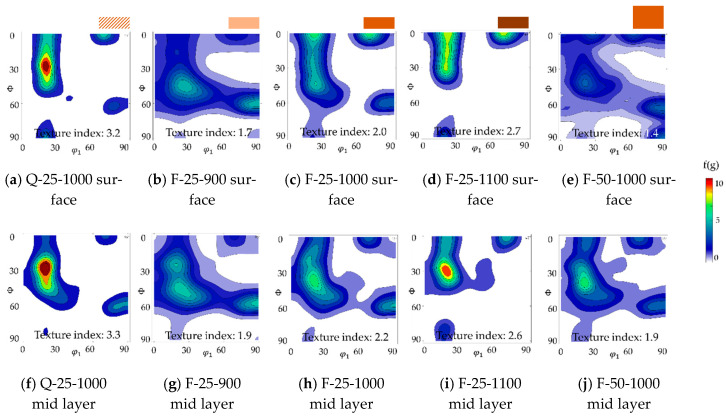
Textures near the surface (**a**–**e**) and in mid layer (**f**–**j**) for annealed sheets: (**a**,**f**) Q-25-1000, (**b**,**g**) F-25-900, (**c**,**h**) F-25-1000, (**d**,**i**) F-25-1100, (**e**,**j**) F-50-1000. Textures are displayed using the ODF φ_2_ = 45° section.

The influence of the annealing temperature on texture is shown in Figure 9b–d,g–i. A trend of higher intensities near the ND-cube components at φ_1_ = 20°/70°, ϕ = 0°, φ_2_ = 45° with higher annealing temperatures can be seen. Moreover, the main and typical recrystallization component φ_1_ = ~20°, ϕ = 45°, φ_2_ = 45° seemed to move to smaller ϕ values and its intensity increased with higher temperatures. Additionally, the second typical recrystallization component φ_1_ = 90°, ϕ = 60°, φ_2_ = 45° vanished at higher temperatures. The mid layer tended to have a lower intensity on the aforementioned ND-cube components and the distribution was not as sharp. It can be presumed that the nucleation and growth rate distribution varied strongly depending on temperature and thickness layer. Therefore, the growth rate distribution was closely linked to the grain boundary mobility distribution. For example, at low temperatures (900 °C), both distributions could be more or less the same resulting in a relatively weak texture with typical components mainly depending on the rolling texture and slightly preferential nucleation. All nuclei have the same chance to form and grow equally fast. At higher temperatures, but below the so-called compensation temperature (where all mobilities are the same), on the other hand, those distributions seem to form maxima and some nuclei arise more often and grow faster resulting in a more textured material and slightly different texture components [42,43,44]. This explanation could also elucidate the trend of abnormal grain growth at high temperatures. However, this hypothesis will require further, more detailed investigation.

The influence of different cold rolling degrees on texture after final annealing is shown in Figure 9c,e,h,j. In the mid layer, the texture of the thin sheet was slightly more concentrated than the texture of the thick sheet (Texture index: 2.2 > 1.9); however, the difference was not pronounced. Near the surface, this difference was a little bit stronger (Texture index: 2.0 > 1.4). Both textures were dominated by the components near φ_1_ = 20°, ϕ = 35°, φ_2_ = 45° and φ_1_ = 90°, ϕ = 55°, φ_2_ = 45°. These two orientations are commonly found in recrystallized textures of electrical steel. Except for these dominant components, two weak ND-cube components at φ_1_ = 25°/65°, ϕ = 0°, φ_2_ = 45° could be observed in both cases as well, which are favorable components with regard to the magnetic properties of rotating machines. This phenomenon was more obvious near the surface of the sheets. Large portions along the λ-fiber formed during rolling prevailed, especially near the surface of the thinner sheet.

The influence of cold rolled, differently cooled hot strips on texture after final annealing is shown in Figure 9a,c,f,h. Within the investigated samples, material Q showed the strongest texture (Texture index: 3.3) with a pronounced peak at φ_1_ = 20°, ϕ = 30°, φ_2_ = 45° associated with the α*-fiber. Although the grain size was in the same range after annealing at 1000 °C, a significant difference in texture appeared. Since nuclei form during deformation and recovery, their orientations are closely related to the rolling texture. Therefore, if the rolling texture is sharp, there is a high probability that the recrystallization texture is also sharp. Material Q-25 annealed at 1000 °C already showed a comparable but slightly sharper texture which was only reached at a final annealing temperature of 1100 °C for F-25. Hot strip Q had a more heterogeneous microstructure with higher stored energy in comparison to hot strip F. After cold rolling, the deformation structure differed as described above. A higher stored energy results in a lower recrystallization start temperature and therefore potentially in an earlier start of grain growth. As demonstrated in this example, the initial hot strip can affect the texture after final annealing giving the possibility to decrease annealing temperature and time.

#### 3.3.3. Influence on the A-Parameter

**Figure 10:**
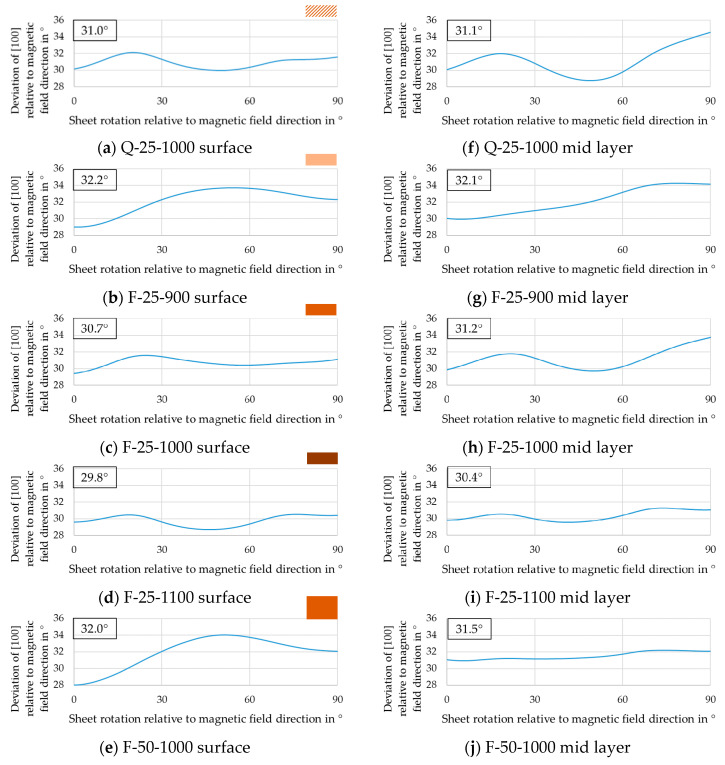
Curve and mean value of the A-parameter near the surface (**a**–**e**) and in the mid layer (**f**–**j**) for annealed sheets: (**a**,**f**) Q-25-1000, (**b**,**g**) F-25-900, (**c**,**h**) F-25-1000, (**d**,**i**) F-25-1100, (**e**,**j**) F-50-1000.

The influence of annealing temperature on the A-parameter is shown in Figure 10b–d,g–i. In this regard, 1100 °C resulted in the flattest curve with the lowest mean A-parameter, which can be correlated with good, isotropic magnetic properties for rotating electric machines.

The influence of different cold rolling degrees on the A-parameter after annealing is shown in Figure 10c,e,h,j. When the direction of the magnetic field coincides with the rolling direction, i.e., if the abscissa is 0°, the A-parameter near the surface is smaller than in the mid layer irrespective of the sheet thickness. The reason is that the texture near the surface is weaker and in addition has a higher proportion of ND-cube (promoting a small A-parameter) than the texture in the mid layer. When the direction of the magnetic field is perpendicular to the rolling direction, that is, when the abscissa is 90°, the A-parameter is larger than at 0° in both sheets and for both thicknesses. This indicates that the orientations present in the annealing texture exacerbate magnetization in the transverse direction. While the A-parameter of F-25-1000 was relatively flat, when the abscissa was around 45°, F-50-1000 showed a local maximum. This could be correlated to the Goss component where a “hard” <111> direction is parallel to the magnetization vector for this abscissa value. Focusing only on the average A-parameter (boxes in Figure 10), the deviation was less than 1° and thus very similar in all cases except for the texture near the surface of the thin sheet, where it still did not exceed 2°. Therefore, if the hot strip contains a high portion of ND-rotated-cube, the cold rolling degree has little effect on the final texture and A-parameter.

The influence of cold rolled, differently cooled hot strips on the A-parameter after annealing is shown in Figure 10a,c,f,h. In the comparison of the two materials Q and F, the A-parameter was alike in value and dependence to magnetization direction. Detailed investigations on the differences of the annealed material in terms of magnetic behavior for all relevant frequencies are summarized in a recent publication [40].

## 4. Discussion

This article analyzes the influence of process parameters during hot rolling, cold rolling, and final annealing on grain size and texture of NO electrical steel to enable a controlled, application-specific determination of beneficial process parameters and hence optimize the final properties. A single alloy with a silicon content of 3.2 wt.-% was used for the entire study. The following section will discuss the results obtained and compare them to existing results for similar alloys from the literature and to previous work conducted on Fe-2.4 wt.-% Si NO electrical steel by the same authors [45,46], wherever possible.

During hot rolling, the final cooling method has a significant effect on microstructure and texture. Quenching the material leads to a banded structure while furnace cooling exhibits a mostly recrystallized grain structure. Concerning the microstructure and texture gradient between near surface and mid layer, the gradient is the result of the macroscopic shear gradient and temperature profile, which occurs during hot rolling. Comparing surface and mid layer, the mid layer is primarily deformed by plain strain deformation at a higher temperature than the surface. Both effects stimulate recovery instead of recrystallization. In contrast, the surface layer experiences shear deformation, which results in the texture components described in Section 3.1. Additionally, the temperature is lower near the surface due to heat losses and the contact with work rolls. The higher effective strain and lower temperature promote recrystallization at the surface layer of the hot strip. Independent of the final cooling method, a texture gradient is present. Nevertheless, in both cases, optimizing the process parameters results in hot strip textures with high intensities of favorable ND-rotated-cube components. Given the fact that the finishing temperatures of 850 °C for hot strip Q and 880 °C for hot strip F are very close to each other, the microstructure of hot strip Q with small equiaxed grains at the surface and bands in the mid layer can be imagined as the initial state for the furnace cooled hot strip. Furnace cooling can be considered a kind of hot strip annealing during which recovery, recrystallization, and grain growth take place and consequently the differences between surface and mid layer after furnace cooling can be explained. Near the surface, small recrystallized grains already exist after the last hot rolling pass which will grow until they touch each other, which then slows down further growth as the driving force originating from stored energy is exhausted, whereas, in the mid layer, the overall stored energy is lower and few nucleation sites are present, leading to larger equiaxed grains and some remaining elongated (not recrystallized) grains after cooling. In comparison to previous research on Fe-2.4 wt.-% Si [46], a larger grain size can be observed for Fe-3.2 wt.-% Si; however, the band or block structure in the mid layer cannot be dissolved completely. For the experiments with Fe-2.4 wt.-% Si, a thin cast slab with a thickness of 64 mm was used. Therefore, a higher total reduction was realized in hot rolling with more reverse rolling passes, inducing more deformation energy and at the same time enabling recrystallization in the mid layer that dissolves the banded structure.

During cold rolling, the initial hot strip state and the cold rolling degree have an impact on the properties. When starting from a banded hot strip structure (strip Q), this cannot be dissolved in cold rolling. However, starting from fully recrystallized grains (strip F), the grains become elongated in rolling direction and shear/deformation bands appear. As the final thickness is reduced, the elongation and shear/deformation bands become more pronounced. Near the surface of the Fe-3.2 wt.-% Si NO electrical steel considered here, a typical rolling texture with α- and γ-fiber is generated after cold rolling as was also observed in an earlier study on Fe-2.4 wt.-% Si NO electrical steel [46]. In the mid layer of the Fe-3.2 wt.-% Si NO electrical steel, however, a large portion of ND-rotated-cube persists after cold rolling for both initial microstructures. This is promising when compared to the results of a previous study on Fe-2.4 wt.-% Si NO electrical steel [47], which showed only unfavorable α- and γ-fiber after cold rolling. It indicates that the ND-rotated-cube present after hot rolling can be partially preserved during cold rolling. In contrast, most commercial electrical steels show high intensity around the α- and γ-fiber after hot rolling, which is further strengthened during cold rolling. The rolling strategies pursued in this paper thus provide a route to improve the final texture and magnetic properties. Additionally, the texture index in the mid layer of the Fe-3.2 wt.-% Si NO electrical steel reduces with further deformation making the sheet more isotropic. This might be an additional explanation for the aforementioned rule of thumb in industry that thinner strips result in favorable magnetic properties.

During final annealing, temperature and cold strip properties are most important for the final grain size and texture. The incubation time before recrystallization, where later growing nuclei can form through recovery processes, is small (<5 s) for thin electrical sheet (high deformation) and high temperatures (>1000 °C). Furthermore, smaller average grain sizes were generally observed near the surface when compared to the mid layer. This can be explained by the surface layer experiencing large shear stresses and thus storing more deformation energy [31]. As a result, more nucleation sites develop and start growing during recrystallization [48]. In addition, those nuclei also grow faster because of the higher driving force correlated to the stored deformation energy. The high nuclei density nuclei get into contact quite quickly and further growth can only occur by grain growth (lower driving force), rather than recrystallization. In the mid layer, fewer nuclei are formed, and the resulting new grains can grow longer without touching each other resulting in a higher final grain size.

A previous study regarding the temperature effect on the grain size of a NO electrical steel with 2.4 wt.-% Si showed that higher annealing temperatures (900 °C vs. 1000 °C) lead to larger grain sizes and relatively weaker γ-fibers [47]. As expected, the current study shows similar results with respect to the influence of temperature. The thermally activated movement of grain boundaries typically results in smaller metastable grain sizes for lower temperatures and reaching the metastable state required more time at lower temperatures. Furthermore, precipitation on grain boundaries can have a huge influence on their mobility and thus on the texture development and final grain size distribution. As this can only be investigated in detail with elaborated transmission electron microscopy or atom probe tomography, it is beyond the scope of this work. The same previous study also showed that the cold rolling degree has a strong influence on the recrystallized texture. Therefore, the initial state for final annealing was a cold strip with a very strong α- and γ-fiber in the mid layer. In the current study, moderate ND-rotated-cube components were present in the initial state before annealing. In comparison, this leads to a reduction in γ-fiber and an increase in ND-cube components with increasing temperatures, while generally resulting in a more concentrated texture. Additionally, when high ND-cube components are already present, the cold rolling degree has little effect on the recrystallized texture.

During magnetization of NO electrical steel, the texture influence is dominant only close to the maximum polarization [49], therefore all process routes considered in this paper (quenching after hot rolling, low cold rolling degree, and different temperatures during final annealing) might prove beneficial for a given application. At lower polarizations, the grain size and energy induced during cold rolling play a dominant role. Especially the grain size is an important factor as it decreases hysteresis losses while increasing excess losses [50]. Thus, the optimal grain size is always application dependent. At low frequencies (wind turbine), the hysteresis losses are dominant, thus a large grain size is needed and at higher frequencies (electric vehicle engines), the excess losses can get quite big, requiring a smaller grain size. Therefore, it is necessary to achieve a magnetic preferable texture with ND-cube components in combination with a grain size matching the application.

## 5. Conclusions

The analysis of grain size and texture along the processing steps of hot rolling, cold rolling, and final annealing of Fe-3.2 wt.-% Si NO electrical steel revealed strong interrelations. On the laboratory scale, hot and cold rolling as well as final annealing experiments were performed to evaluate the impact of different process parameters, such as hot strip cooling conditions, total cold rolling reduction, and final annealing temperature. By varying the process parameters, different microstructures, grain size distributions, and textures were produced. For the final annealed state, the A-parameter was considered to estimate the possible magnetic properties. Based on this comprehensive study on Fe-3.2 wt.-% Si NO electrical steel, the following conclusions can be drawn:

- Substantial shares of favorable ND-cube components were generated in hot rolling. Therefore, a mainly rotated ND-cube texture was achieved via a low hot strip thickness of 1 mm and finishing temperatures of 880 °C and 850 °C in combination with quenching and furnace cooling, respectively.
- These favorable ND-cube texture components can be partially preserved in cold rolling. This is easier for quenched hot strips with a partially banded microstructure than for furnace cooled strips with more homogeneous microstructure.
- Generally, the annealed texture is weaker than the cold rolled texture. Only annealing at 1100 °C, which includes grain growth, strengthens the texture with maxima at φ_1_ = 20°, ϕ = 35°, φ_2_ = 45°.
- As expected, grain size is inversely proportional to the cold rolling degree and always differs between surface and mid layer. However, after final annealing this difference reduces for reduced sheet thickness.
- Annealing at 1100 °C results in the lowest mean A-parameter with the flattest curve, which correlates with good, isotropic magnetic properties for rotating electric machines. However, the A-parameter provides no information about the frequency dependence of the magnetic properties of the produced material and thus the grain size influence should be considered additionally.

Based on the findings in this study future research should focus on ways to preserve even more ND-cube components through the processing chain and particularly also during final annealing.

## Figures and Tables

**Table 1:** Chemical composition of the investigated Fe-3.2 wt.-% Si NO electrical steel. Measurement by spark spectral analysis (S, N, P, and C are either overestimated or below the detection limit, thus the maximum content based on supplier information (*) is provided).

Chemical Element	C	Mn	Si	Al	S	N	P	Fe
Weight Percent (wt.-%)	* 0.002	0.17	3.16	0.89	* 0.003	* 0.006	* 0.07	balance

**Table 2:** Hot rolling schedule.

Hot Rolling Pass Number	1	2	3	4	5	6	7
	Reverse Rolling			Continuous Rolling			
Final thickness in mm	15.0	7.5	3.9	2.1	1.3	1.15	1.0
Reduction in %	55	50	48	46	38	12	13
Roll diameter in mm	340	340	340	340	200	200	195

**Table 3:** Cold rolling schedule: cold strip of 0.50 mm in 3 passes and cold strip of 0.25 mm in 6 passes.

Cold Rolling Pass Number	1	2	3	4	5	6
Final thickness in mm	0.75	0.57	0.48	0.33	0.26	0.25
Reduction in %	25	24	16	31	21	4

**Table 4:** Samples after final annealing and their corresponding processing description.

Name.	Description		
	Hot Rolling	Cold Rolling	Final Annealing
Q-25-1000	1 mm, water quenching	0.25 mm	1000 °C, 1 min
F-25-900	1 mm, furnace cooling	0.25 mm	900 °C, 2 min
F-25-1000	1 mm, furnace cooling	0.25 mm	1000 °C, 1 min
F-25-1100	1 mm, furnace cooling	0.25 mm	1100 °C, 1 min
F-50-1000	1 mm, furnace cooling	0.50 mm	1000 °C, 1 min

**Table 5:** Mean grain size of all annealed sheets categorized by material state and layer.

		Name				
		Q-25-1000	F-25-900	F-25-1000	F-25-1100	F-50-1000
Sheet layer	surface	156.55 µm ± 103.59 µm	28 µm ± 17.42 µm	152.89 µm ± 92.85 µm	164.09 µm ± 98.12 µm	86.42 µm ± 55.39 µm
	mid layer	161.27 µm ± 97.32 µm	34.04 µm ± 19.38 µm	138.58 µm ± 82.28 µm	174.04 µm ± 95.55 µm	117.46 µm ± 72.5 µm

## Data Availability

The data are available from the corresponding author upon reasonable request.
